# Inflammasome activation in airway epithelial cells after multi-walled carbon nanotube exposure mediates a profibrotic response in lung fibroblasts

**DOI:** 10.1186/1743-8977-11-28

**Published:** 2014-06-10

**Authors:** Salik Hussain, Stacey Sangtian, Shamika M Anderson, Ryan J Snyder, Jamie D Marshburn, Annette B Rice, James C Bonner, Stavros Garantziotis

**Affiliations:** 1 Clinical Research Unit, National Institute of Environmental Health Sciences (NIEHS)/National Institute of Health (NIH), Research Triangle Park, Durham, NC, USA; 2 Department of Biological Sciences, North Carolina State University, Raleigh, NC, USA

**Keywords:** Multi-walled carbon nanotubes, Nanotoxicity, Fibrosis, Pyroptosis, NLRP3 inflammasome, Human bronchial epithelia

## Abstract

**Background:**

*In vivo* studies have demonstrated the ability of multi-walled carbon nanotubes (MWCNT) to induce airway remodeling, a key feature of chronic respiratory diseases like asthma and chronic obstructive pulmonary disease. However, the mechanism leading to remodeling is poorly understood. Particularly, there is limited insight about the role of airway epithelial injury in these changes.

**Objectives:**

We investigated the mechanism of MWCNT-induced primary human bronchial epithelial (HBE) cell injury and its contribution in inducing a profibrotic response.

**Methods:**

Primary HBE cells were exposed to thoroughly characterized MWCNTs (1.5-24 μg/mL equivalent to 0.37-6.0 μg/cm^2^) and MRC-5 human lung fibroblasts were exposed to 1:4 diluted conditioned medium from these cells. Flow cytometry, ELISA, immunostainings/immunoblots and PCR analyses were employed to study cellular mechanisms.

**Results:**

MWCNT induced NLRP3 inflammasome dependent pyroptosis in HBE cells in a time- and dose-dependent manner. Cell death and cytokine production were significantly reduced by antioxidants, siRNA to NLRP3, a caspase-1 inhibitor (z-WEHD-FMK) or a cathepsin B inhibitor (CA-074Me). Conditioned medium from MWCNT-treated HBE cells induced significant increase in mRNA expression of pro-fibrotic markers (TIMP-1, Tenascin-C, Procollagen 1, and Osteopontin) in human lung fibroblasts, without a concomitant change in expression of TGF-beta. Induction of pro-fibrotic markers was significantly reduced when IL-1β, IL-18 and IL-8 neutralizing antibodies were added to the conditioned medium or when conditioned medium from NLRP3 siRNA transfected HBE cells was used.

**Conclusions:**

Taken together these results demonstrate induction of a NLRP3 inflammasome dependent but TGF-beta independent pro-fibrotic response after MWCNT exposure.

## Background

Multi-walled carbon nanotubes (MWCNT) exhibit unique electrical, mechanical, thermal and optic properties, which make them the material of choice for a variety of industrial and consumer product applications. Apart from their conventional utilizations in electronics, composite materials and optics, more recent applications of MWCNT include biomedical engineering, biosensors, drug delivery and gene therapy [1-5]. Given their tremendous potential, it is expected that their applications will continue to grow over the coming years [6]. However, some of the properties which make MWCNTs a material of choice from an engineering aspect (e.g. high tensile strength, high aspect ratio and biopersistence) can also lead to potential toxicity in biological systems [7-12].

At present there is no definitive proof of any human disease due to occupational or environmental exposure to MWCNTs. However, a large body of literature show potential toxic effects in rodents including acute lung inflammation, granuloma formation, epithelial-mesenchymal transition and fibrosis [8,13-17]. Moreover, studies have shown that MWCNTs can translocate from the lungs to the pleura where they cause injury to the mesothelial lining and therefore may cause pleural disease [16,18-21]. An excellent review about the pulmonary toxicity of MWCNT can be found elsewhere [22]. These findings raise concerns about the safety of MWCNTs and warrant further in-depth mechanistic analyses to ensure the safety of workers and consumers. Indeed, recent estimates indicate that more than 6 million workers will be involved with nanotechnology by 2020, one third of whom will reside in the US [23].

Airway remodeling, including increased deposition of extracellular matrix proteins (ECM) plays significant role in the development of symptoms associated with lung function decline in asthma and COPD [24,25]. In lungs more than 10% of the total ECM is deposited/degraded each day and fibrosis is actually a disturbed balance in favour of its accumulation either due to excessive production or impaired degradation mechanisms [26]. MWCNT induce airway remodeling and fibrosis in rodent models after respiratory exposures [13,15,16]. However, the mechanisms of such effects remain poorly elucidated. Some *in vitro* studies have postulated the role of Transforming Growth Factor-beta (TGF-β) production in the induction of pro-fibrotic response after MWCNT exposures [8,13]. Still others, using cell lines, postulated the role of epithelial-mesenchymal transformation (EMT) in airway fibrosis [17,27]. These studies mainly focussed on the role of lung macrophages as key mediators in airway fibrosis in rodent models and did not address the injury to airway epithelia as a contributor to these responses. Using a relevant translational model, we explore the mechanistic pathway of primary human epithelial injury and its contribution in airway remodeling after MWCNT exposures. We report here that MWCNT induce pyroptosis (caspase-1-dependent inflammatory cell death) in primary human bronchial epithelial cells. Furthermore, we demonstrate a novel pro-fibrotic mechanism after MWCNT exposures of primary human bronchial epithelial (HBE) cells, which involves nucleotide-binding oligomerization domain (NOD)-like receptor protein 3 (NLRP3) inflammasome activation in HBE cells, inducing Tenascin-C (TN-C), Osteopontin (OPN), Tissue Inhibitor of Metalloprotease-1 (TIMP-1) and Procollagen-1 (PC-1) expression, and proliferation in fibroblasts. Moreover, we demonstrate that this process occurs without de-novo TGF-β expression and can be effectively modulated by siRNA inhibition of epithelial NLRP3 activation.

## Results

### Characterization of nanomaterials and their suspensions

**Figure 1:**
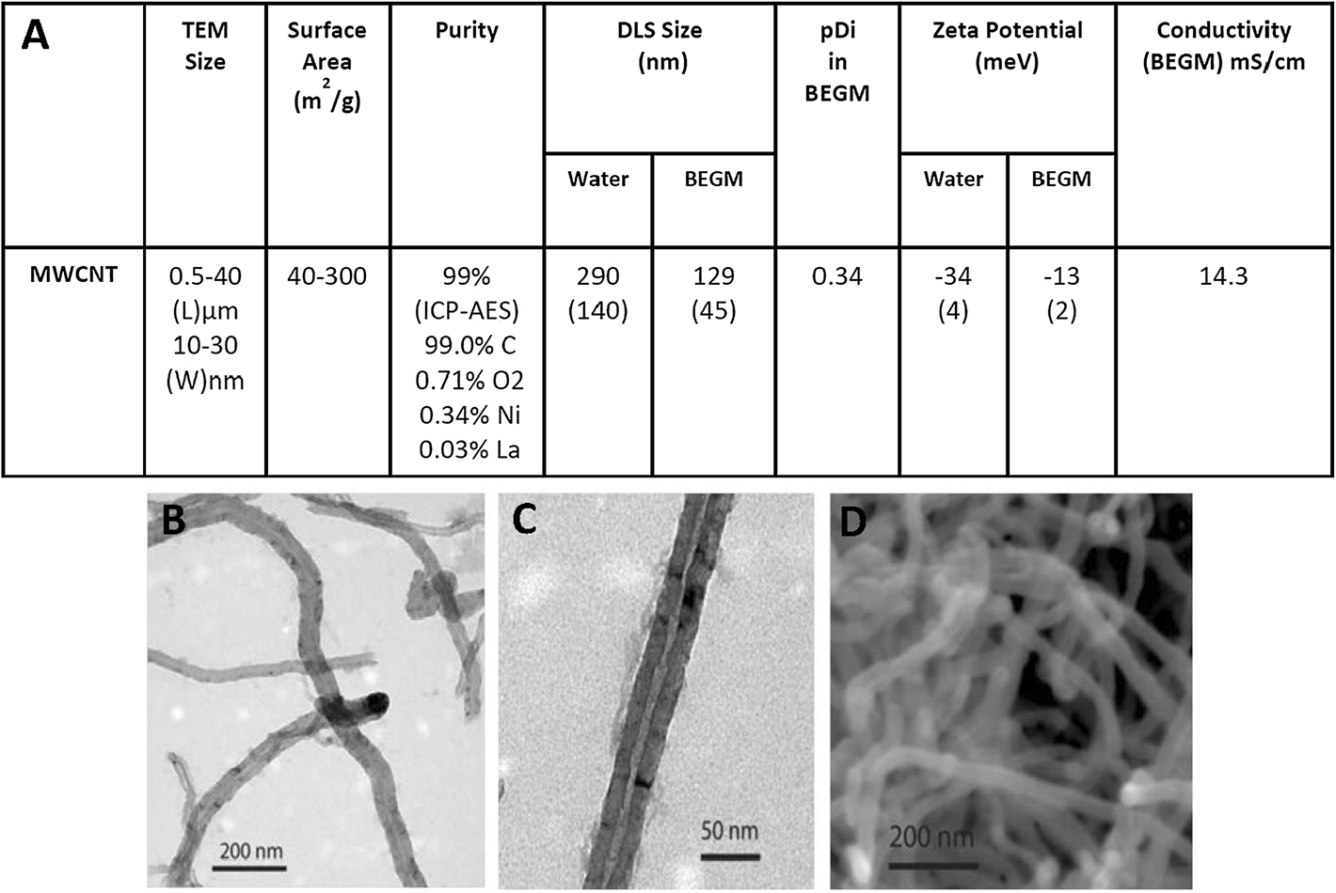
**Physico-chemical characteristics of MWCNT powder and suspensions. A)** summary of characteristics in a table format. pDi refer to polydispersity index **B**, **C)** TEM and **D)** SEM analyses of MWCNT powder.

Detailed physico-chemical characterization of MWCNTs was performed before cell culture testing. Data provided by the manufacturer were verified by an independent source and have been reported previously [18]. Briefly, the ICP-AES measurements by the independent source showed 99% elemental carbon, 0.34% Ni and 0.03% La and 0.7% O_2_ (Figure 1). These nanotubes have 10–30 nm average external diameters, 0.5-40 μm average length and 109.29 m^2^/g BET surface area. High resolution TEM and SEM images of MWCNT dry powder is given in Figure 1. MWCNT were suspended in cell culture medium (24 μg/mL) and suspensions were analyzed for hydrodynamic diameter and zeta potentials using DLS technique. DLS revealed that MWCNT form aggregates of 129 ± 45 nm size (Additional file 1). Carbon Black (CB) NPs and Min-u-Sil showed 95 ± 10 nm and 363 ± 64 nm hydrodynamic diameters respectively. Zeta potential analyses revealed −13 ± 2 mV, −12 ± 3 mV and −8 ± 5 mV values for MWCNT, CB and Min-U-Sil respectively. MWCNT and CB samples were tested free of bacterial endotoxin contamination (<0.3 EU/mL) by endpoint chromogenic limulus amebocyte assay (LAL assay).

### MWCNTs enter HBE cells and cause ultra-structural damage

MWCNTs were taken up by primary HBE cells and were found either in vesicles or free inside the cytoplasm (Additional file 2). In most instances MWCNT clumps/bundles were seen inside vesicles (Additional file 2B and C), however, in some cases we found single nanotubes in contact with the cell membrane or in vesicular membranes, which appeared to be piercing through the membrane (Additional file 2D).

### MWCNT induce NLRP3 inflammasome dependent pyroptosis

**Figure 2:**
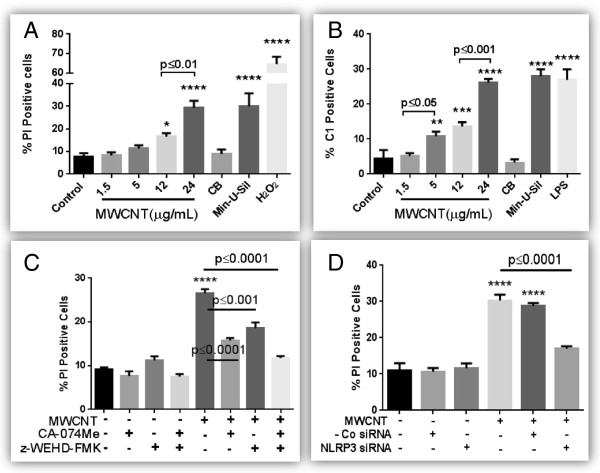
**MWCNT induce pyroptosis in HBE cells. A)** A dose response of MWCNT (1.5-24 μg/mL equivalents 0.37-6.0 μg/cm^2^) induced cell death after 24 hours exposure. Cells were analyzed by flow cytometry after labeling with PI. CB (24 μg/mL) and Min-U-Sil (100 μg/mL) were used as negative and positive particle controls respectively. H_2_O_2_ (1 mM) was used as positive control for oxidative stress induced cells death. **B)** Caspase-1 activation in HBE cells after 24 hours MWCNT exposure, analyzed by flow cytometry. CB (24 μg/mL) was used as negative control while Min-U-Sil (24 μg/mL) and LPS (1 μg/mL) were used as positive controls. **C)** Protection of cell death using specific inhibitors for cathepsin B (CA-074ME, 10 μM) and caspase-1 (Z-WEHD-FMK, 50 μM). Cells were pre-treated (for 1 hour) with these inhibitors before treatment with MWCNT (24 μg/mL) in the presence of inhibitor for further 24 hours and analyzed by flow cytometry. **D)** Modulation of cell death by specific siRNA against NLRP3 inflammasome. Transfection of HBE cells was performed using either specific siRNA against NLRP3 inflammasome or a nonspecific negative control siRNA. Cells were treated with MWCNT (24 μg/mL) for 24 hours. Cell death was measured by flow cytometry after labeling with PI. Data were analyzed by analysis of variance (ANOVA) followed by Tukey’s post hoc test. Graphs show average ± SEM of three independent experiments with triplicate of each conditions, *p < 0.05, **p < 0.01, ***p < 0.001, ****p < 0.0001 (between media-treated control and treatments).

MWCNT exposure at 12 and 24 μg/mL induced significant cell death after 24 hours of exposure (flow cytometery data) (Figure 2A). No cell death was observed after CB NP exposure (shape control/negative control) while Min-U-Sil (particulate positive control) did cause significant toxicity. Hydrogen peroxide (H_2_O_2_) was used as positive control for oxidative stress. A kinetic analysis (4, 18, 24 and 48 hours exposure) revealed significant cell death induction starting at 18 hours with 24 μg/mL MWCNT exposure and at 24 hours with 12 μg/ml MWCNT exposure with the response being persistent up to 48 hours (Additional file 3). Cell death estimation was verified using three independent methods i.e. flow cytometry (propidium iodide staining), LDH assay and trypan blue exclusion cell counts). All three assays for cytotoxicity estimation demonstrated comparable results (Additional file 4A-B). Furthermore, MWCNT exposure caused a significant increase in caspase-1 positive cells (Figure 2B). These results were also confirmed using western blot analysis for total and cleaved caspase 1 (Additional file 5). A significant increase in cathepsin B release from HBE cells was also confirmed by immunohistochemistry (Additional file 6). Treatment of HBE cells with caspase-1 inhibitor (z-WEHD-FMK) and lysosomal protease cathepsin B inhibitor (CA- 074Me) either alone or in combination significantly protected the cells from MWCNT-induced toxicity (Figure 2C). Further, we demonstrated that siRNA against NLRP3 significantly reduced cell death (Figure 2D) of HBE cells confirming its role in the cytotoxic responses induced by MWCNTs.

### Mechanism of MWCNT-induced inflammatory response

**Figure 3:**
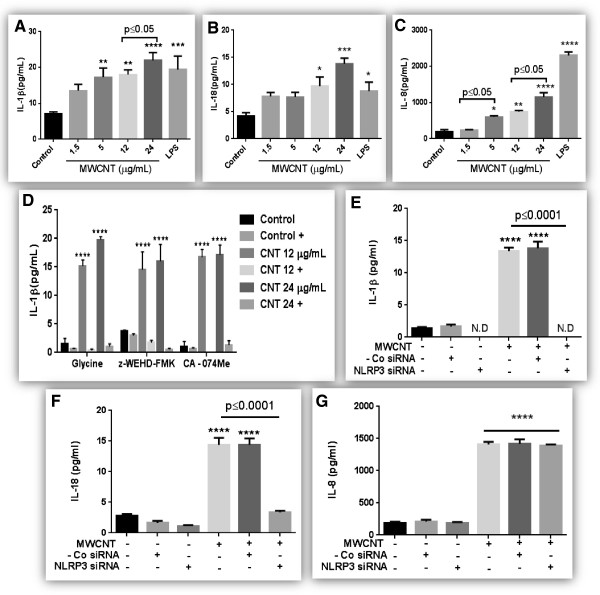
**Mechanism of MWCNT induced pro-inflammatory response in HBE cells. A-C)** A dose response of cytokine secretion (IL-1β, IL-18, IL-8) in cell culture supernatants of HBE cells exposed to MWCNT (1.5-24 μg/mL) for 24 hours. LPS (1 μg/mL) was used as positive control. Quantity of cytokines in cell culture supernatants was estimated using commercially available ELISA/Bioplex kits. **D)** Modulation of IL-1β production after pretreatment with pharmacological inhibitors of caspase-1 (Z-WEHD-FMK) and cathepsin B (CA-074Me) or pore blocking agent (glycine). Cells were pre-treated with these inhibitors as described in Materials and methods and levels of IL-1β in cell culture supernatants were quantified by commercially available ELISA. **E-G)** Modulation of IL-1β, IL-18 and IL-8 production by specific siRNA against NLRP3 inflammasome. Data were analyzed by analysis of variance (ANOVA) followed by Tukey’s post hoc test. Graphs show average ± SEM of three independent experiments with triplicate of each condition, *p < 0.05, **p < 0.01 and ***p < 0.001, ****p < 0.0001 (between media-treated control and treatment).

A significant increase in the release of inflammatory mediators (IL-1β, IL-18 and IL-8) in cell culture supernatants was observed after 24 hours of MWCNT exposure (Figure 3A-C). An increase in the levels of IL-1β and IL-8 were observed even at non-cytotoxic dose (with 2 out of three tested methods) suggesting an inflammatory response in living cells, as opposed to cytokine release from ruptured cells. However, no significant change in the levels of GRO-α, IP-10, IL-6, and IL-12 was observed, while IFN-γ, MIP-1α, RANTES and TNF-α were only detected in control LPS treated cells (data not shown). A time course (16, 24 and 48 hours) of inflammatory mediator production after MWCNT exposure is presented in Additional file 7. MWCNT-induced increase in IL-1β production was significantly reduced after pre-treatment with glycine (an inhibitor of pore formation), or caspase-1 and cathepsin B inhibitors (Figure 3D). Further, we demonstrated that siRNA against NLRP3 significantly reduced IL-1β production (Figure 3E) by HBE cells confirming the role of NLRP3 inflammasome in the cytotoxic and inflammatory responses induced by MWCNT. We also measured levels of IL-18 and IL-8 in the supernatants of NLRP3 transfected cells and confirmed that IL-18 production was indeed inflammasome dependent (Figure 3F) while IL-8 production is not effected by NLRP3 knockout (Figure 3G).

### ROS production and its role in MWCNT-induced toxicity

**Figure 4:**
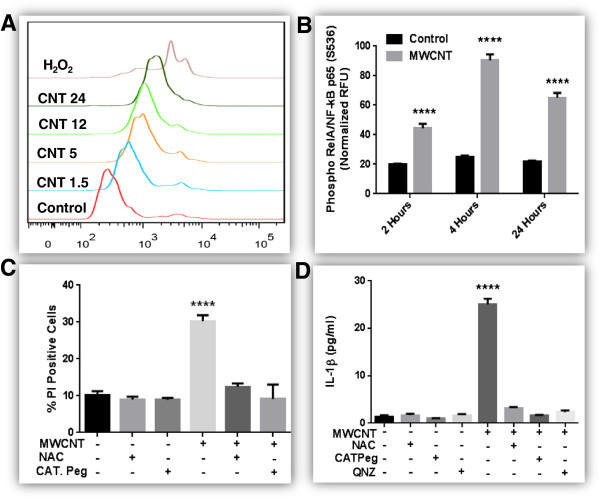
**Role of ROS production in MWCNT induced toxicity. A)** Dose response of ROS production. HBE cells were treated with different doses of MWCNT (1.5-24 μg/mL equivalents 0.37-6.0 μg/cm^2^) for 24 hours. Hydrogen peroxide (1 mM) was used as positive control. ROS were labeled with HE and analyzed by flow cytometry. At least 10,000 cells/events excluding debris were captured. **B)** Time course analysis of NF-κB (REL A/P65) phosphorylation in HBE cells after exposure to 24 μg/mL MWCNT for different time points. Cells based ELISA was performed according to manufacturers recommendations. **C)** Modulation of MWCNT induced cell death with antioxidants (NAC and CAT Peg). Cells were pre-treated with 5 mM NAC or CAT Peg (1000 I.U) (30 minutes) and then exposed to MWCNT (24 μg/mL) for 24 hours in the presence of antioxidants. Cell death analysis was performed using flow cytometry. **D)** Modulation of MWCNT induced IL-1β production with antioxidants (NAC and CAT Peg) and NF-κB inhibitor (QNZ). Cells were pre-treated with 5 mM NAC, CAT Peg (1000 I.U) (30 minutes) or QNZ (10 nM) and then exposed to MWCNT (24 μg/mL) for 24 hours in the presence of inhibitors. Cytokine production was analyzed by commercially available ELISA. Data were analyzed by analysis of variance (ANOVA) followed by Tukey’s post hoc test. Graphs show average ± SEM of three independent experiments with triplicate of each condition, *p < 0.05, **p < 0.01, ***p < 0.001, ****p < 0.0001 (between media-treated control and treatment).

We observed significant dose and time-dependent production of ROS after exposure to MWCNT (Figure 4A). Time dependent phosphorylation of nuclear factor-kappa B (NF-κB) was also observed after MWCNT exposure (Figure 4B). NF-κB phosphorylation, loss of mitochondrial membrane potential (∆Ψm) and lipid peroxidation after MWCNT exposures were significantly reduced after utilizing antioxidants (N-acetylCysteine (NAC) and Catalase poly ethylene glycol (Cat Peg)) (Additional file 8A-C). Cytotoxic effects of MWCNT were successfully abolished using NAC or Cat peg (Figure 4C). Moreover, IL-1β production was completely inhibited by QNZ (NF- κB inhibitor) in addition to NAC and Cat peg (Figure 4D). It is noteworthy that ROS and inflammasome activation are well-established triggers for inflammasome assembly and cytokine production and all the known triggers of NLRP3 activation are ROS inducers [28].

### Conditioned medium from MWCNT-treated HBE cells induce fibroblast proliferation and Pro-fibrotic gene expression

**Figure 5:**
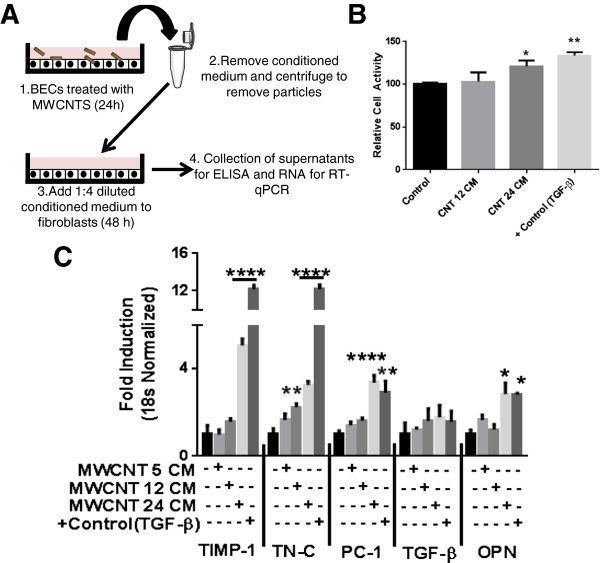
**HBE cells conditioned medium induces fibroblast proliferation and profibrotic gene expression. A)** Experimental protocol for conditioned media experimentation. HBE cells were treated with different doses of MWCNT (12, 24 μg/mL equivalents 3.6 μg/cm^2^) for 24 hours, cell culture supernatants were removed, centrifuged and diluted 1:4 with DMEM/F-12 medium (without serum). Fibroblasts were grown till near confluence and serum starved for 24 hours then exposed to conditioned medium from epithelial cells for 48 hours. mRNA was extracted from these cells to perform pro-fibrotic gene expression analysis using real time quantitative RT-PCR. **B)** Fibroblast proliferation assay after CM exposure for 48 hours. TGF-β (10 ng/mL) was used as a positive control. **C)** Dose response analysis of TIMP-1, TN-C, PC-1, TGF-β and OPN expression in MRC-5 cells after CM exposures. Data were analyzed by analysis of variance (ANOVA) followed by Tukey’s post hoc test. Graphs show average ± SEM of three independent experiments with triplicate of each condition, *p < 0.05, **p < 0.01, ***p < 0.001, ****p < 0.0001 (between media-treated control and treatment).

**Figure 6:**
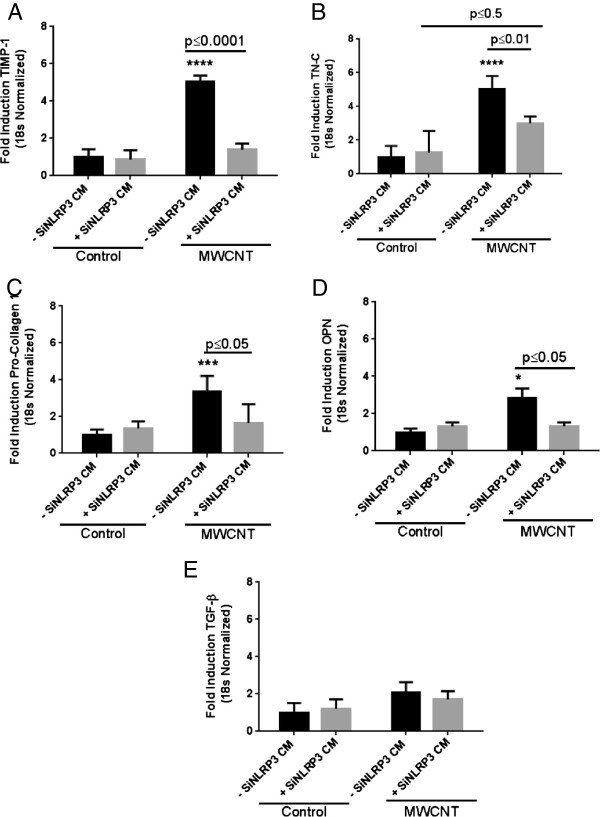
**Role of NLRP3 inflammasome activation in HBE cells in inducing a pro-fibrotic gene expression in fibroblasts. A-E)** Modulation of TIMP-1, TN-C, PC-1, OPN and TGF-β using conditioned media from HBE cells transfected with siRNA against NLRP-3 inflammasome. Data were analyzed by analysis of variance (ANOVA) followed by Tukey’s post hoc test. Graphs show average ± SEM of three independent experiments with triplicate of each conditions, *p < 0.05, **p < 0.01, ***p < 0.001, ****p < 0.0001 (between media-treated control and treatment).

**Figure 7:**
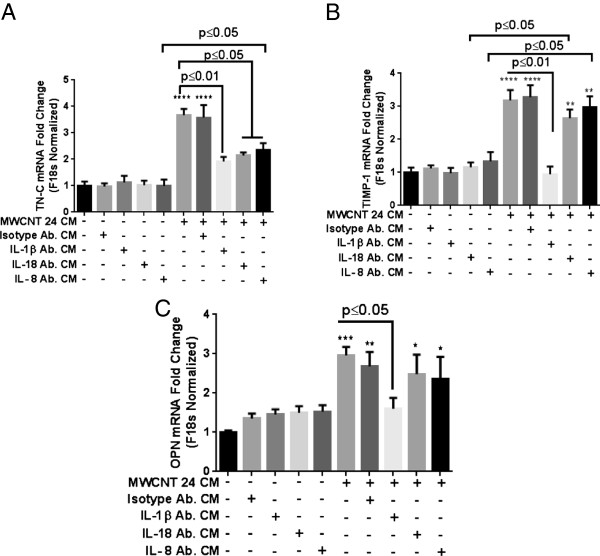
**Role of inflammatory cytokines in conditioned medium from MWCNT treated HBE cells in inducing pro-fibrotic gene expression.** Conditioned medium from MWCNT-treated epithelial cells (24 hour exposure at 24 μg/mL) was incubated with neutralization antibody against IL-1β (5 μg/mL), IL-18 (5 μg/mL), or IL-8 (10 μg/mL) at 37°C for 30 minutes before adding to the MRC-5 cells. Gene expression of **A)** TN-C **B)** TIMP-1 and **C)** OPN after 48 hours of exposure was evaluated by real time quantities RT-PCR. Data were analyzed by analysis of variance (ANOVA) followed by Tukey’s post hoc test. Graphs show average ± SEM of three independent experiments with triplicate of each conditions, *p < 0.05, **p < 0.01, ***p < 0.001, ****p < 0.0001 (between media-treated control and treatment).

Pro-fibrotic response was confirmed by evaluating fibroblast proliferation by a recently validated *in vitro* assay and measuring gene expression for TIMP-1, TN-C, PC1 and OPN. A conditioned media (CM) approach was developed to analyze the role of epithelial derived factors in fibroblast gene expression as co-culturing of epithelial cells and fibroblasts was not technically possible (Figure 5A). An increase in fibroblast proliferation was observed after exposure to CM from MWCNT treated HBE cells (Figure 5B). We observed a significant increase in fibroblast gene expression of TIMP-1, TN-C, PC1 and OPN after CM exposure for 48 h (Figure 5C). TGF-β, a known inducer of fibrotic response was used as a positive control. It is noteworthy that we did not observe an increase in gene expression of TGF-β itself after exposure to conditioned medium from epithelial cells treated with MWCNT (Figure 5C). The pro-fibrotic gene expression in fibroblasts depended on NLRP3 inflammasome activation in HBE Cells, since a significant decrease in the expression of TIMP-1, TN-C, PC1 and OPN was observed when CM from HBE cells transfected with NLRP-3 siRNA was used (Figure 6A-D). Again CM from NLRP3 siRNA transfected cells did not lead to any change in gene expression of TGF-β (Figure 6E). The role of IL-1β, IL-18 and IL-8 was established in the observed responses utilizing neutralizing antibodies against them (Figure 7A-C). IL-1β played a significant role in the induction of tested pro-fibrotic markers (TN-C, TIMP-1 and OPN). Interestingly, all cytokines appear to contribute in the induction of TN-C as their neutralization afforded only partial protection.

## Discussion

**Figure 8:**
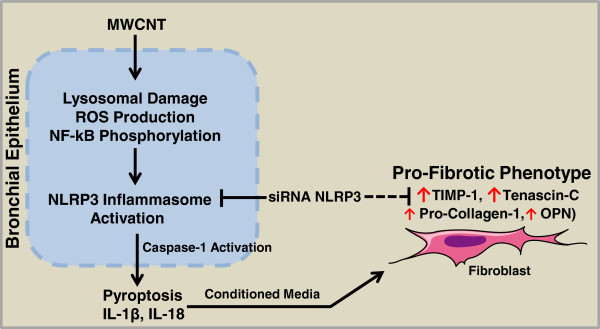
**MWCNT-induced cell death (pyroptosis) mechanisms in primary HBE cells and its contribution in pro-fibrotic gene expression in fibroblasts.** Internalization of MWCNT in HBE cells leads to lysosomal damage, release of cathepsin B, ROS production, NF-κB phosphorylation, NLRP3 inflammasome activation and their inhibition can successfully abolish MWCNT-induced toxicity. NLRP3 inflammasome derived factors from HBE cells induce pro-fibrotic (TN-C, TIMP-1, PC-1 and OPN) gene expression in MRC-5 cells.

In the present study we mechanistically investigated MWCNT-induced cell injury and elaborated its role in the induction of a profibrotic response. We found that MWCNT induce pyroptosis and inflammasome activation in primary human bronchial epithelial cells. Furthermore, we describe a mechanism through which profibrotic gene expression is induced in fibroblasts in response to inflammasome-derived factors from injured bronchial epithelial cells. An overview of all these mechanisms is presented in Figure 8. To the best of our knowledge this is first report confirming inflammasome dependent pro-fibrotic response after MWCNT exposures to the primary HBE cells.

Some literature reports have discussed inflammasome activation in LPS stimulated myeloid cells (mostly cell lines) after nanotube exposures [29,30]. Myeloid cells are known to produce enormous amounts of cytokines after endotoxin stimulation, however it has always been difficult to establish the relevance of the large amounts of endotoxin used *in vitro* with realistic *in vivo* exposures. Our study differs from above mentioned work in four important aspects: 1) We used primary human bronchial epithelial cells; 2) We uncovered the molecular mechanism of pyroptosis in HBE cells; 3) We observed inflammasome activation without prior priming with LPS; and 4) We uncovered the biological significance of the inflammasome activation after MWCNT exposure as a trigger for a pro-fibrotic response. Moreover, it is important to note that we observed the pro-fibrotic response without significant induction of TGF-β which has been shown to be induced after MWCNT exposure in rodent studies as well as studies with cell lines [8,13]. This indicates a potential limitation of these models to predict human exposures/responses after MWCNT exposure.

Growing evidence confirms a significant contribution of airway epithelium in pulmonary immune responses to inhaled pollutants/allergens [31,32]. It has been shown that epithelium-derived factors significantly contribute in the pathogenesis of a variety of respiratory disorders like asthma, idiopathic pulmonary fibrosis and chronic obstructive pulmonary disease [33,34]. This study further confirms the significance of primary human epithelial cells as contributors to the innate immune response, as we observed a significant production of inflammatory mediators from these cells even after non-cytotoxic MWCNT exposures. An exuberant activation of epithelial cells can lead to an uncontrolled response and thus could have deleterious consequences. We further attempted to activate epithelial cells with LPS to see if cytotoxic responses increase further. Our data indicate these cells are stimulated to their maximum and cannot be further stimulated (Additional file 9A). This confirms that production of low amount of cytokines is indeed a strategy by the body to avoid uncontrolled immune response. However, it is worth mentioning that even the low amounts of inflammatory mediators produced by epithelial cells have significant biological impact and can lead to fibroblast proliferation and pro-fibrotic gene expression. We observed no significant effect of LPS priming on the release of IL-1β and IL-18 from BECs (Additional file 9B-C). These findings are in line with recently reported observations from silica exposed bronchial cells [35]. Interestingly, we observed significant increase in the production of IL-8 after LPS priming (Additional file 9C). This on one hand confirms differential signaling cascades for IL-8 production, and on the other hand further affirms that no increase in IL-β was a true biological response and could not be attributed to LPS.

Pro-fibrotic response is characterized by increased fibroblast proliferation and excessive ECM deposition either due to excessive production or impaired turnover. We studied expression of important components of airway remodeling i.e. TN-C, OPN, PC-1 and TIMP-1. We demonstrate here a significant increase in expression of these markers after exposure to conditioned medium from MWCNT treated HBE cells. Increased levels of OPN, PC-1 and TIMP-1 are noted in rodent lungs in bleomycin induced pulmonary fibrosis [36-38]. TN-C is prototypic family member of Tenascin family of extracellular matrix glycoproteins. TN-C is highly expressed under pathological conditions such as inflammation, infection, tumorigenesis and wound healing [39]. Recently, it has been shown that TN-C deficiency attenuates TGF-β-mediated fibrosis following rodent lung injury [40]. In patients with asthmatic inflammation enhanced TN-C expression correlates with grade of bronchial inflammation of mast cells and eosinophils [41]. TIMPs inhibit the activity of matrix metalloproteinases and thus play and important role in maintaining a balance between ECM deposition and degradation. TIMP-1 is an important member of the TIMP family and has known association with different disease processes in human airways. Increased TIMP-1 levels associated with airflow obstruction were found in sputum from subjects with asthma and chronic bronchitis [42]. It was noted that decreased MMP-9/TIMP-1 ratio lead to an increase in airway wall thickness causing pro-fibrotic response through increased matrix deposition [43]. It is worth mentioning that transcriptomic analysis revealed TIMP-1 induction in mouse lungs after MWCNT instillation, however monocultures of mouse lung epithelial cells did not show an increase in TIMP-1 mRNA [44]. This further confirms the need to study cellular interactions to accurately describe the effects of nanomaterials. Our results of increased mRNA expression of OPN are in line with reports describing its over expression after particle and fibre exposures and in granulomatous lung diseases [45,46]. OPN is a matricellular protein mediator which is upregulated in patients of idiopathic pulmonary fibrosis [47]. Increased levels of OPN cause fibroblast proliferation, migration and adhesion [48]. Taken together, these literature findings when combined with our expression data indicate a plausible mechanism of airway remodeling after exposure to conditioned medium from MWCNT treated HBE cells.

We also characterized the role of NLRP3 inflammasome activation in HBE cells as a modulator of the pro-fibrotic response in fibroblasts. NLRP3 inflammasome activation has been shown in various mammalian cell types (macrophages, dendritic cells, monocytes, epithelial cells, fibroblasts, keratinocytes and neurons) in response to diverse stimuli, including microbes, viral RNA, ATP, uric acid crystals, environmental particles and fibres [29,49-53]. Among various members of the inflammasome family, NLRP3 has shown to be particularly responsive to danger-associated molecular patterns (DAMPS) and fibres and particles including nanomaterials in addition to detecting pathogen associated molecular patterns (PAMPS) [50,54]. Previously, it was reported that MWCNT-induced macrophage activation promotes pulmonary fibrosis through the TGF-β/Smad pathway [8]. Using primary human cells we describe here a novel mechanism through which epithelial cells can initiate a profibrotic response through activation of the NLRP3 inflammasome. Recently, cristobalite silica induced inflammasome activation in epithelial cells was shown to induce fibroblast proliferation [35]. Moreover, it was demonstrated that NLRP3 and ASC deficient mice did not develop pulmonary fibrosis after bleomycin exposure whereas wild type mice had increased collagen deposition the lungs [55]. Taken together the results demonstrate that the NLRP3 inflammasome can orchestrate fibrosis in both sterile and pathogen-driven conditions [56]. The NLRP3 inflammasome has been implicated in the pathogenesis of various disorders like asbestosis, silicosis, gout, Alzheimer’s disease and diabetes, mostly through the production of IL-1β [49,50,53,57]. We demonstrate here that IL-1β present in conditioned medium from epithelial cells plays a significant role in inducing pro-fibrotic gene expression. Indeed, IL-1β is known to induce TIMP-1 expression through regulation of AP-1and/or NF-κB in fibroblasts [58]. Moreover, increased expression of OPN is described after exposure to inflammatory cytokine such as IL-1β [59]. Interestingly, we show that IL-1β as well as IL-18 and IL-8 contribute in the induction of TN-C. It is worth mentioning that conditioned medium from HBE cells did not induce toxicity or ROS production in fibroblasts, thus ruling out oxidative stress as an effector mechanism for fibroblast proliferation or pro-fibrotic gene expression (Additional file 10). Taken together these results clearly demonstrate that inflammasome downstream (IL-1β and IL-18) as well as inflammasome independent IL-8 contributes in the pro-fibrotic response after MWCNT exposure. This gene expression appear to be mainly derived through IL-1β (neutralization reduces expression of TIMP-1, OPN and TN-C) whereas other cytokines (IL-18 and IL-8) appear to have only minor role (neutralization only protects partially from TN-C overexpression).

The NLRP-3 inflammasome is well known platform for the activation of caspase-1 which is driven by induced proximity. Indeed, in addition to its role in activation of IL-1β and IL-18, overt inflammasome activation can also lead to cell death through caspase-1 (Pyroptosis) [60]. Our results are in line with recent reports describing caspase-1 activation in HBE cells or myeloid cells (monocytes and macrophages) after exposure to carbon black nanoparticles, silver nanoparticles, cristobalite silica particles, carbon nanotubes and cholesterol crystals [29,30,35,61,62]. Our finding of caspase-1 activation after nanotube exposure without priming with LPS is in line with recent literature describing caspase-1 activation after silica exposure in epithelial cells [35]. However, using primary HBE cells, we have additionally explored the expression of pro-fibrotic genes and verified the role of different cytokines using specific neutralizing antibodies in MWCNT induced pro-fibrotic response. Moreover, we demonstrate that the pro-fibrotic gene expression occur without de novo TGF-beta expression. We did not find any increase in caspase 3/7 or RIP 1 levels, thus confirming the cell death modality as pyroptosis and not apoptosis or necroptosis (Additional file 11). Moreover, we demonstrate here that inflammasome activation and resulting IL-1β secretion occur without priming by TLR ligation. These results are in agreement with recent findings that silica particles and particulate matter exposure can lead to inflammasome activation without LPs priming [35,54]. NLRP3 inflammasome activation is sometimes described as multistep process, requiring TLR priming to increase expression of pro IL-1beta and IL-18 (through NF-kB phosphorylation) before actual inflammasome assembly. However, various other triggers e.g. oxidative stress can also activate NF-kB leading to increase production of these cytokines. It is an ongoing debate whether NLRP3 inflammasome activation absolutely require TLR-agonist priming. For instance, it has been shown that NLRP3 assembly and resulting IL-1β secretion can occur after exposure to various stimuli without engaging TLR receptors [63-65]. These studies propose various alternate pathways to explain inflammasome assembly without TLR priming includes activation through danger associated molecular patterns (DAMPS), P2X7 receptor, serum amyloid A.

## Conclusions and perspectives

In conclusion, the results of present study elucidate the significance of NLRP3 inflammasome activation in epithelial cells as a key mediator of pro-fibrotic gene expression in MRC-5 cells. Moreover, these results indicate that MWCNT-induced bronchial epithelial injury shares common mechanisms with various well-known respiratory pathologies, such as asthma, COPD, and idiopathic pulmonary fibrosis. This further raises the possibility of modulation of pre-existing disorders by nanotubes exposure in susceptible human populations (already shown to occur in rodent models). Lastly, this study further confirms the significance of primary human epithelial cells as contributors towards the innate immune response. Further studies to elucidate the role of differentiation in cell injury mechanisms, using *ex-vivo* differentiated bronchial epithelial cells at air-liquid interfaces, are currently in progress in our laboratory. More in depth studies using translational approaches are needed for further clarifying the human health impacts of nanotubes exposures.

## Materials and methods

### Nanomaterials and characterization

Commercially available MWCNT (0.5-40 μm length and 10–30 nm external diameter), prepared by chemical vapor deposition (CVD) method, were utilized in this study (Helix Material Solutions, Inc., Richardson, TX). More details about physical characteristics of the bulk materials used in this study can be found in supporting information. Carbon black (CB) NPs of 90 nm diameter (99.9% pure, spherical shape) were used as chemical composition negative control and were purchased from Evonik (formerly Degussa). Crystalline Silica particles (Min-U-Sil (99.2% pure α-Quartz) were used as particulate positive control (US Silica Company, Berkeley, CA). These materials have been used extensively for *in vitro* and *in vivo* exposures to study the toxicity nanomaterials and their characteristics have already been described [18,66,67].

### Nanomaterial suspensions

Stock suspensions of nanomaterials were made at 2 mg/mL concentration in BEGM media containing 0.01 mg/mL Dipalmitoylphosphatidylcholine (DPPC) and 0.6 mg/mL Bovine serum Albumin (BSA), both from Sigma Aldrich. Studies were conducted to know the impact of these dispersants on the viability, mitochondrial activity and inflammation in HBE cells. These results are presented in Additional file 12. Our results confirmed no harmful effect of these additives on the studied parameters at all the tested doses. This dispersion protocol is already described to yield maximum suspension stability and mimic more closely the *in vivo* lung surfactant fluid [13]. All exposure suspensions were freshly prepared from this stock solution after sonication for 12 minutes at 235 W (20 s pulses with a 5 s pause) using a Mesonix S 4000 cuphorn sonicator (Qsonica LLC, Newtown, CT, USA). After sonication nanomaterials were suspended in cell culture medium and used to expose cells within 5 min after vortexing. Dynamic light scattering analysis was performed to evaluate size distribution and zeta potentials of nanomaterial suspensions in cell culture medium using ZetaSizer Nano (Malvern Instruments, Westborough, MA, USA) as described by us previously [68]. Electrophoretic mobility was converted into zeta potential using the Helmholtz-Smoluchowski equation.

### Cells and culture conditions

*HBE cell*s were purchased from Lonza (Walkersville, MD USA) under the trade name of Normal Human Bronchial Epithelial Cells. Cells from 5 individuals were utilized in the present study and all experiments were performed between passage 3–4. These cells were cultivated in BEGM media (BEBM media supplemented with 5 μg/mL insulin, 0.5 ng/mL hEGF, 0.5 μg/mL hydrocortisone, 0.5 μg/mL epinephrine, 50 μg/mL gentamycin, 50 μg/mL amphoteracin B, 10 μg/mL transferrin, 6.5 ng/mL triidotyronin, 0.13 mg/mL bovine pituitary extract (all supplied by Lonza) and 100 μg/mL penicillin and streptomycin (Sigma Aldrich). Cells were cultivated on non coated plastic material (recommended by the supplier) at a density of 20,000 cells per cm^2^ and kept in an incubator at 37°C, 5% CO_2_, and 95% relative humidity. Cells were exposed to nanomaterials after 72 hours of culture (nearly 90% confluence). In the experiments involving pharmacological inhibitors, cells were pre-treated with fresh media containing inhibitors for desired period of time and then treated with NP suspensions in the presence of inhibitors. Following inhibitors were used in different experiments: z-WEHD-FMK (Caspase-1 inhibitor) (R and D Systems, Minneapolis MN), CA-074Me (Cathepsin B inhibitor) (Sigma-Aldrich, St. Louis, MO), Staurosporine (apoptosis positive control) (Sigma-Aldrich, St. Louis, MO).

For cytokine neutralization assays, cells were incubated for 30 minutes with neutralizing antibodies (anti IL-1β, anti IL-8 (both from R and D Systems, Minneapolis MN) or anti IL-18 (MBL, Japan) before treatment with MWCNT (in the presence of antibodies) for further 24 hours.

*MRC-5 fibroblasts* were purchased from American Type Culture Collection (ATCC) and maintained in DMEM/F12 medium supplemented with 10% Fetal Calf Serum and 100 μg/mL penicillin and streptomycin. Recently, it was demonstrated that these cells respond to nanoparticle exposures in a similar way as primary human lung fibroblasts [69]. Cells were cultured in 12 well plates till confluence and then serum starved for 24 hours. Cells were exposed to a mixture of 1 part CM (from nanotubes treated HBE cells) and 4 parts DMEM/F12 medium for 48 hours. At the end of exposure period cells were lysed to collect mRNA for real time RT-PCR analyses. TGF-β (10 ng/mL) was used as positive control in for fibrosis experiments. We first attempted co-culturing primary HBE cells and MRC-5 fibroblast cells. However, this approach was not successful as either medium required for optimal growth of one type of cells causes either death or changed morphology of the other cell type. Our experimentation indicated that primary HBE cells are very sensitive to serum and media other than BEGM. We observed that serum containing DMEM (even as low as 10%) results in changes in morphology and causes cell death (data not shown). On the other hand fibroblasts react to the addition of BEGM with morphological changes, altered metabolic activity and cell death (data not shown). We therefore adapted a conditioned medium approach to overcome this hurdle. It is noteworthy that this is also a more physiologically relevant approach as epithelial cells are directly exposed to nanomaterials while fibroblasts are mostly exposed to epithelial secretions and only come in contact with nanomaterials in case of damaged/denuded epithelia. This approach has already been reported elsewhere to study the toxicity of silica particles [35]. We verified that CM did not introduce toxicity and ROS production in fibroblasts (Additional file 10).

### Transmission electron microscopy

HBE cells were grown in two chamber cell culture slides till sub-confluence and treated with different concentrations of MWCNT for 24 h. At the end of treatment period, cells were fixed in 3% glutaraldehyde and processed for TEM analysis as described previously [68]. Thin sections (80 nm) were cut, placed on formvar copper grids and stained with uranyl acetate and lead citrate. After staining, the sections were examined on a FEI Tecnai 110 kV microscope at 80 kV, and digital photomicrographs were taken.

### Validation of *In vitro* assays

Moreover, necessary measures (using alternate methods and controls) were taken to avoid artifacts introduced by the nanomaterials in spectrophotometric and florescent assays. Cytokine ELISA assays were standardized by using an approach described previously [70]. Known amounts of cytokine standards were incubated with different nanotubes concentrations to verify possible adsorption of cytokines on nanotubes. Particle only controls were also run in parallel to verify the impact of particles on absorbance measurements. Cytotoxicity results were verified using three independent techniques i.e. LDH assay, trypan blue exclusion cell counts and flow cytometry using propidium iodide. We observed significant impact of nanotube concentrations higher than 24 μg/mL on absorbance (data not shown). We used approaches described recently in detail to avoid interferences with flow cytometry assays (PI and ROS production) [71]. Particles were incubated with fluorochromes to rule out their binding onto particle surfaces. No uptake of PI/HE was observed for any type of particles used in this study. We observed that flow cytometry was least impacted by the nanotubes and it gave most statistically reliable readings in terms of highest number of cell counts per conditions.

### Flow cytometry

Cell death was quantified in MWCNT treated cells by flow cytometery using propidium Iodide (PI) probe which detects the integrity of cell membrane. Briefly, supernatants were collected at the end of treatment period and cells were trypsinated using 0.025% trypsin-EDTA (Invitrogen). The action of trypsin was inhibited using 10% fetal bovine serum (Sigma Aldrich, St. Louis, MO, USA) and cells were mixed with respective supernatant samples. Cells were centrifuged for 5 minutes at 400 g and resuspended in cell culture media containing PI (2.5 μg/mL final concentration). Analysis was performed using BD FACSAria II equipment at 488 nm excitation and 615 nm emission wavelengths. After eliminating cell debris at least 10000 cells were analyzed to determine the percentage of PI positive cells.

### Caspase-1 assay

Caspase-1 activation in HBE cells was quantified using FAM-FLICA™ Caspase-1 Assay (FAM-YVAD-FMK) kit (Immunochemistry Technologies, Bloomington, MN). Cells were labelled according to manufacturers recommendation and analysis was performed using BD FACSAria II equipment. After eliminating cell debris at least 10000 cells were analyzed to determine the percentage of capase-1 positive cells.

### Cytokine analysis

The concentrations of IL-1 β, IL-18, IL-8, MCP-1 and IL-13 released into the culture supernatant after 16, 24 or 48 hours MWCNT exposure were evaluated with either commercially available human enzyme-linked immunosorbent assay (ELISA) kit (R&D Systems, Minneapolis MN) or BD Bioplex assay system (BD Biosciences) according to manufacturers recommendations.

### siRNA for NLRP3

Cells were transfected using Lipofectamine 2000 reagent (Block-iT Transfection Kit, Invitrogen) according to manufacturer’s recommendation and incubated with specific 100 nM siRNAs for 24 hours prior to nanomaterial exposures. Silencer select® siRNA against NLRP3 (sense 5′- > 3′ GCUUUGUCCUCGGUACUCAtt and UGAGUACCGAGGACAAAGCtg, Silencer select® negative control #2 siRNA (product number 4390846 Ambion®) and BLOCK-iT™ Green Fluorescent Oligo for Lipid Transfection (transfection control, Invitrogen) were used. Inflammasome down regulation was verified with RT- qPCR and NLRP3 protein analyses (Additional file 13).

### ROS production

ROS production was estimated by flow cytometery after staining with Hydroethidine (HE) probe as described by us previously [72]. After eliminating cell debris at least 10000 cells were analyzed to determine the percentage of HE positive cells. The graphic presentation of results was created using FlowJo Software (TreeStar Inc, Ashland, OR).

### Phospho REL A/p65 NF-κB ELISA

Cell based human Phospho-Rel A/p65 (S536) immunoassay was performed according to manufacturers recommendations (R and D Systems Minneapolis MN). Assay results were validated using immunohistochemistry for phospho p65 (data not shown) and by analyzing necessary controls as mentioned in cytokine estimation assay.

### Fibroblast proliferation assay

Fibroblast proliferation was assessed 24 hours after exposure to MWCNT by a recently validated *in vitro* assay described in detail elsewhere [73]. We used 10 ng/mL TGF-β as a positive control.

### Real time RT-PCR

Total RNA was collected from adherent cell cultures using RNEasy Midi Kit (Qiagen, Germantown, MD) according to manufacturer’s recommendations. Amount of RNA was quantified using NanoDrop (Thermo Fisher Scientific, USA). Reverse transcription (RT) reaction to obtain cDNA was performed with SuperScript III kit and oligo dTs (Invitrogen) exactly following manufacturer’s recommendations. Final PCR reaction volume was 25 μL containing Power SYBR Green Master Mix (Applied Biosciences), 1 μg of cDNA and 500 nM of each specific forward and reverse primer (Table 1). Data was collected on a Stratagene Mx3005P real-time PCR instrument (Agilent, CA, USA). Changes in expression of target genes were determined by ddCt analysis, comparing expression to Ribosomal Protein (18 s) and normalizing to media control. Each fold expression represents an average of at least 3–5 biological replicates per treatment group. Following primers were used for different genes.

**Table 1:** Primer sequences used in the present study for qPCR analyses

**Gene**	**Primers**	**Sequences (5′ → 3′)**
18 s	Forward	GTAACCCGTTGAACCCCATT
	Reverse	CCATCCAATCGGTAGTAGCG
OPN	Forward	CTGAAACCCACAGCCACA
	Reverse	TGTGGAATTCACGGCTGA
TN-C	Forward	AGGGCAAGTGCGTAAATGGAG
	Reverse	TGGGCAGATTTCACGGCTG
TIMP-1	Forward	AGACCTACACTGTTGGCTGTGAG
	Reverse	GACTGGAAGCCCTTTTCAGAG
PC-1	Forward	CCAGAAGAACTGGTACATCAGCAA
	Reverse	CGCCATACTCGAACTGGAATC
TGF-β	Forward	ACGCACCCTGTCTGACTACA
	Reverse	ACCTCTAAGACGGAGCACCA
NLRP3	Forward	GGAGAGACCTTTATGAGAAAGCAA
	Reverse	GCTGTCTTCCTGGCATATCACA

### Immunocytochemistry (ICH)

ICH analysis was performed to evaluate Cathepsin B release and phosphorylation and nuclear translocation of RELA/P65 subunit of NF-κB. Detailed method for the labelling has been published [72]. Following primary antibodies were employed: phosphor-NF-κB p65 (Ser536) (93H1) rabbit monoclonal antibody (Cell Signalling) (1:100), anti-cathepsin B (CB59-4B11,Sigma-Aldrich) (1:100). In case of non-conjugated primary antibodies, Alexa Fluor 488® (1:500) was used as secondary antibody and nuclei were counterstained with Hoechst 33342 stain (1 μg/mL). Images were captured using a Zeiss-Axiovert 40 CFL microscope and processed using Image J software (NIH, USA).

### Western blot analysis

Caspase-1 and RIP-1 levels (total and cleaved) in MWCNT treated cells were evaluated 24 hours post exposure by immune blots. Briefly, proteins were collected from scrapped HBE cells using a mixture of RIPA buffer (Thermo Scientific) and protease inhibitor cocktail (Sigma Aldrich). Proteins were quantified using micro BCA (bicinchoninic acid) assay (Thermo Scientific), separated electrophoretically on a 4-12% bis-tris polyacrylamide gel and transferred to polyvinylidene difluoride (PVDF) membrane. Membranes were blocked using 3% BSA and incubated with 1:500 dilution of primary rabbit monoclonal antibodies (RIP#3493 and casapse-1#2225, Cell Signalling) overnight at 4°C. Membrane was extensively washed after incubation and anti –rabbit HRP-conjugated secondary antibody (1:10,000) (Cell Signalling) was applied for 2 hours. After washing, chemiluminescent signal was developed using ECL Prime (Thermo Scientific) and detected using GBox digital imaging system (SynGene, Frederick MD). Densitometric analysis was performed using Gene-Sys densitometry software (SynGene, Frederick MD) and signal was normalized to α/β tubulin (#2148, Cell Signalling) (1:1000).

### Mitochondrial membrane potential (∆Ψ)

Mitochondrial membrane potential dynamics were evaluated by measuring ΔΨm with mitochondrial selective probe JC-1 (Invitrogen) according to manufacturer’s recommendations. JC-1 is mitochondrial selective probe which accumulates in the mitochondria in potential dependent manner and show a fluorescence emission shift from green (~529) to red (~590). Cells with damaged mitochondria show higher green fluorescence than red. After desired incubation periods, cells were stained and live cell images were taken using Zeiss-Axiovert 40 CFL microscope and processed using Image J software (NIH, USA).

### Lipid peroxidation

Lipid peroxidation was estimated in MWCNT treated cells using Click-iT® Lipid Peroxidation Imaging Kit - Alexa Fluor® 488 (Molecular Probes®) according to manufacturers recommendations. Fluorescence images were taken using Zeiss-Axiovert 40 CFL microscope and processed using Image J software (NIH, USA).

### Statistical analysis

Data are presented as Mean ± SEM from at least three independent experiments with triplicate of each condition during these repeats. Data distribution was analyzed by D’Agostino-Pearson omnibus normality test. Data were analyzed using one-way or two-way Analysis of Variance (ANOVA) followed by Tukey’s post hoc test for multiple comparisons using Graphpad Prism software Version 6.0 (Graphpad Software Inc. San Diego CA). Differences were considered statistically significant at *p* < 0.05 (two-tailed).

## Competing interest

No competing financial interests.

## Authors’ contribution

SH conceived the study, designed and performed experiments, analyzed data and wrote manuscript. SS, SMA, RJS, JDM and AR assisted in experimental work and read manuscript. JCB provided carbon nanotubes and characterization data, read the manuscript and provided intellectual input. SG supervised work, read the manuscript and provided intellectual input throughout the study. All authors read and approved the manuscript.

## Supplementary Material

Additional file 1 Size distribution (DLS Analyses) of nanomaterials used. Click here for file

Additional file 2 TEM analysis of MWCNT uptake by HBE cells after 24 hours exposure. Click here for file

Additional file 3 Kinetics of cytotoxicity in HBE cells after MWCNT exposure. Click here for file

Additional file 4 **LDH Release and Trypan Blue Exclusion counts for toxicity estimation.** A) a dose response of LDH release in the cell culture supernatants after MWCNT exposure for 24 hours. LDH positive control (1:5000) dilution was incubated with nanotubes (24 μg/ml) for 24 hours and measured to check for interference with LDH in addition to using particle only controls for absorbance artifact. B) trypan blue exclusion counts. Data were analyzed by analysis of variance (ANOVA) followed by Tukey’s post hoc test. Graphs show average ± SEM of three independent experiments with triplicate of each condition, *p < 0.05, ***p < 0.001, ****p < 0.0001 (between media-treated control and treatments). Click here for file

Additional file 5 **Dose response analysis of caspase-1 (total and cleaved/active form) in HBE cells after MWCNT exposure for 24 hours.** Tubulin was used as loading control. Click here for file

Additional file 6 **Immunocytochemistry for Cathepsin B in HBE cells after exposure to MWCNT (24 μg/mL) for 24 hours.** Nuclei were counter stained with Hoechst. Images are representative of 3 independent experiments done in duplicate. Click here for file

Additional file 7 Time course for inflammatory cytokine production. Click here for file

Additional file 8 **A) Modulation of NF-κB (p65) phosphorylation, B) mitochondrial membrane potential changes and C) lipid peroxidation assessment using antioxidants (NAC and Cat Peg).** Data were analyzed by analysis of variance (ANOVA) followed by Tukey’s post hoc test. Graphs show average ± SEM of three independent experiments with triplicate of each condition, ****p < 0.0001(between media-treated control and treatments). Images are representative of 3 independent experiments done in duplicate. Click here for file

Additional file 9 **Modulation of cytotoxicity and inflammation after LPS pre-stimulation of HBE cells.** Cells were pre-stimulated with 1 μg/mL LPS for 2 hours and then treated with MWCNT (12 or 24 μg/mL) for 24 hours. A) cytotoxicity analysis by PI labelling followed by flow cytometery. B-D) inflammatory cytokine (IL-1β, IL-18, IL-8) production measured by ELISA. Data were analyzed by analysis of variance (ANOVA) followed by Tukey’s post hoc test. Graphs show average ± SEM of three independent experiments with triplicate of each condition, **p < 0.01, ****p < 0.0001 (between media-treated control and treatments). Click here for file

Additional file 10 **Effects of MWCNT treated HBE cell conditioned medium on A) cytotoxicity (PI analysis) and 2) ROS production (DHE analysis) from fibroblasts using flow cytometry.** MRC-5 medium (DMEM/F12) alone was also used in parallel to know the non-specific effects of conditioned medium. Data were analyzed by analysis of variance (ANOVA) followed by Tukey’s post hoc test. Graphs show average ± SEM of three independent experiments with triplicate of each condition, ****p < 0.0001 (between media-treated control and treatments). Click here for file

Additional file 11 **Analysis of other modalities (apoptosis and necroptosis) of cell death.** A) A time course analysis for caspase 3/7 activation after MWCNT exposure using flow cytometery. Staurosporine (10 μM) was used as a positive control. B) Western blot analysis for necroptosis protein RIP1. Data were analyzed by analysis of variance (ANOVA) followed by Tukey’s post hoc test. Graphs show average ± SEM of three independent experiments with triplicate of each condition, ****p < 0.0001 (between media-treated control and treatments). Click here for file

Additional file 12 Effect of media dispersants (BSA/DPPC) on the A) toxicity B) metabolic activity C) IL-1β production by HBE cells. Click here for file

Additional file 13 **Efficiency of NLRP3 SiRNA knockout A) real time RT-qPCR analysis B) immunostaining of HBE cells with our without NLRP3 SiRNA and with isotype control antibody.** Graph shows average ± SEM of three independent experiments with triplicate of each condition, **p < 0.01 (between control SiRNA and NLRP3 SiRNA, Students *t*-test). Click here for file
