# Supplementary figures and images for: Inflammasome activation in airway epithelial cells after multi-walled carbon nanotube exposure mediates a profibrotic response in lung fibroblasts
Source: Part Fibre Toxicol. 2014 Jun 10;11:28. doi: 10.1186/1743-8977-11-28 (PMC4067690; doi:10.1186/1743-8977-11-28)

## Additional File 1

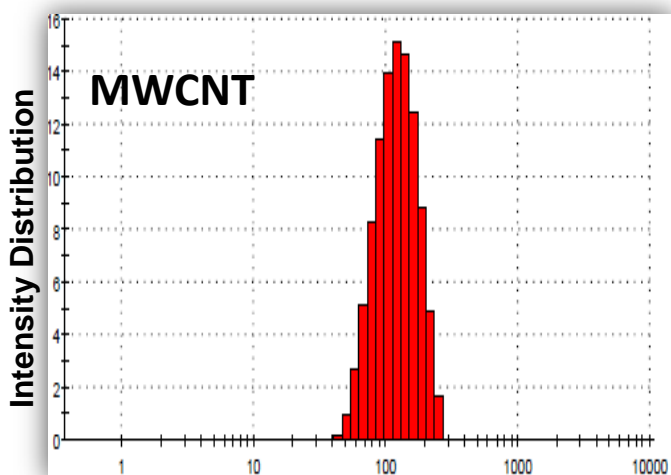

Hydrodynamic Diameter

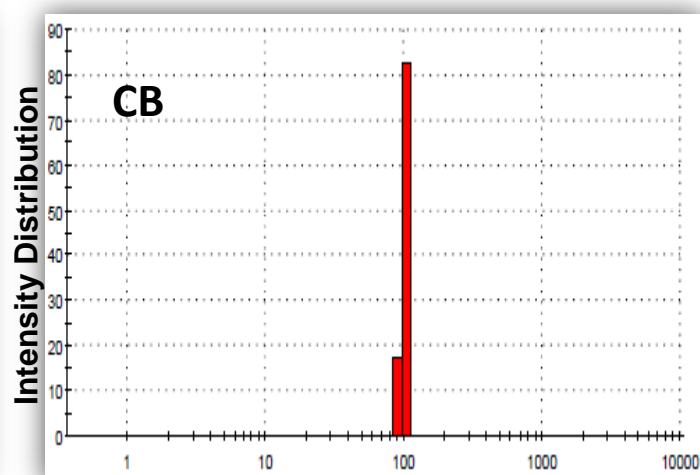

Hydrodynamic Diameter

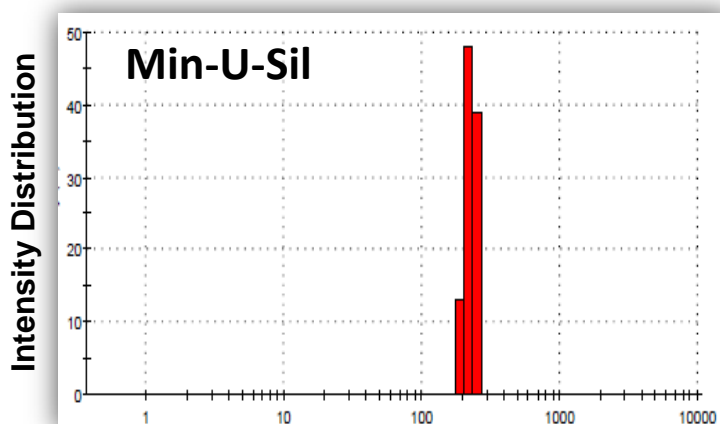

Hydrodynamic Diameter

Supplement: Additional file 1 — Size distribution (DLS Analyses) of nanomaterials used. [file 1743-8977-11-28-S1.pdf]

## Additional File 2

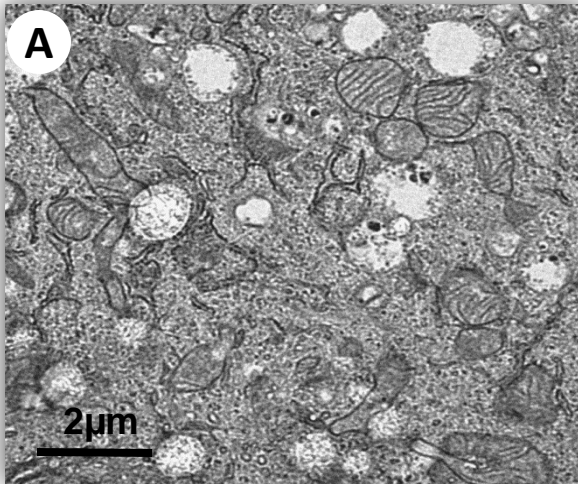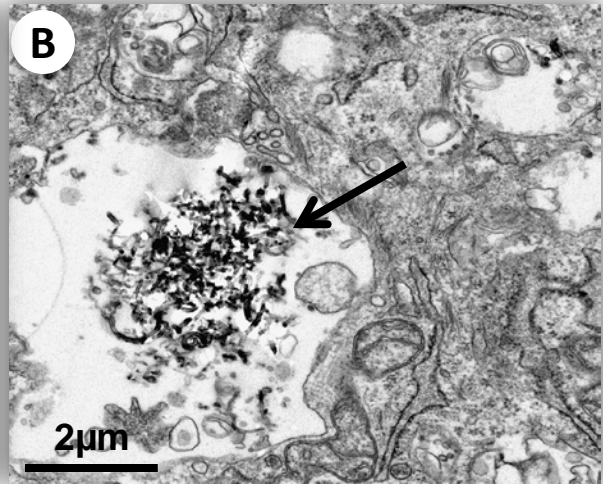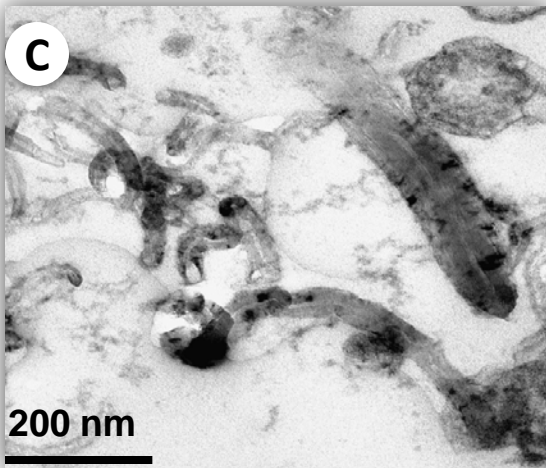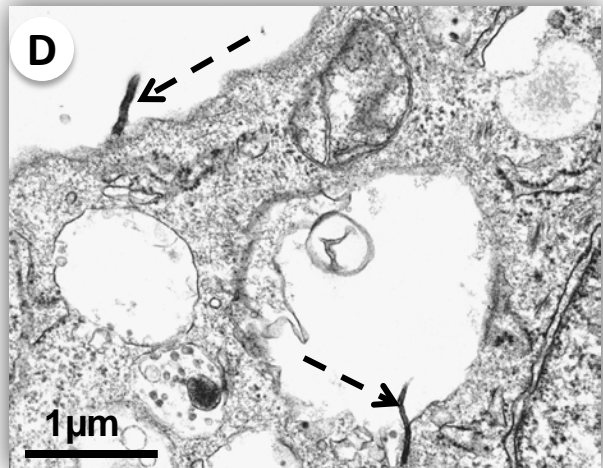

Supplement: Additional file 2 — TEM analysis of MWCNT uptake by HBE cells after 24 hours exposure. [file 1743-8977-11-28-S2.pdf]

## Additional File 3

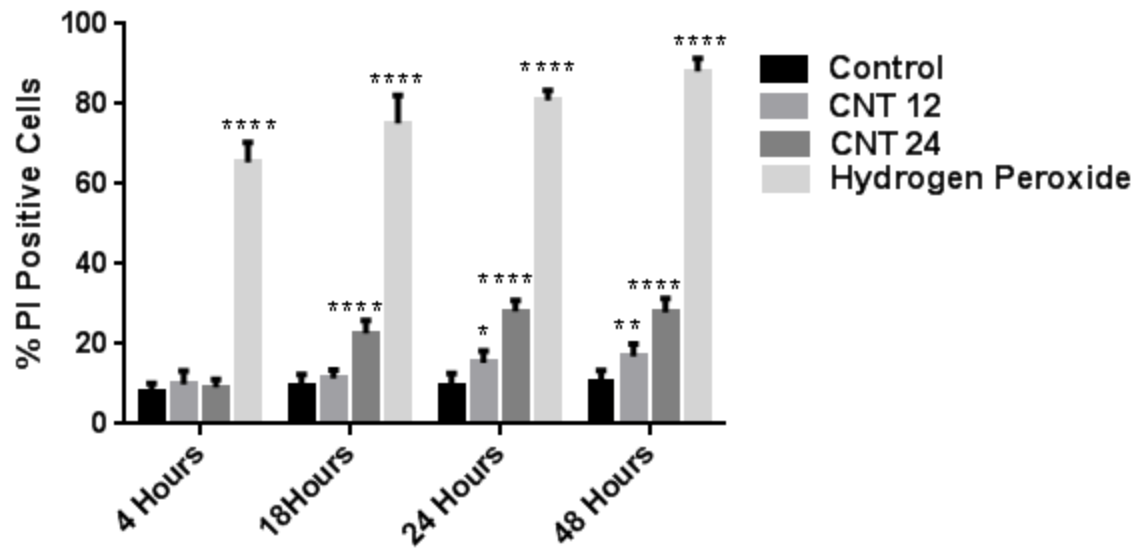

Supplement: Additional file 3 — Kinetics of cytotoxicity in HBE cells after MWCNT exposure. [file 1743-8977-11-28-S3.pdf]

## Additional File 4

**A**

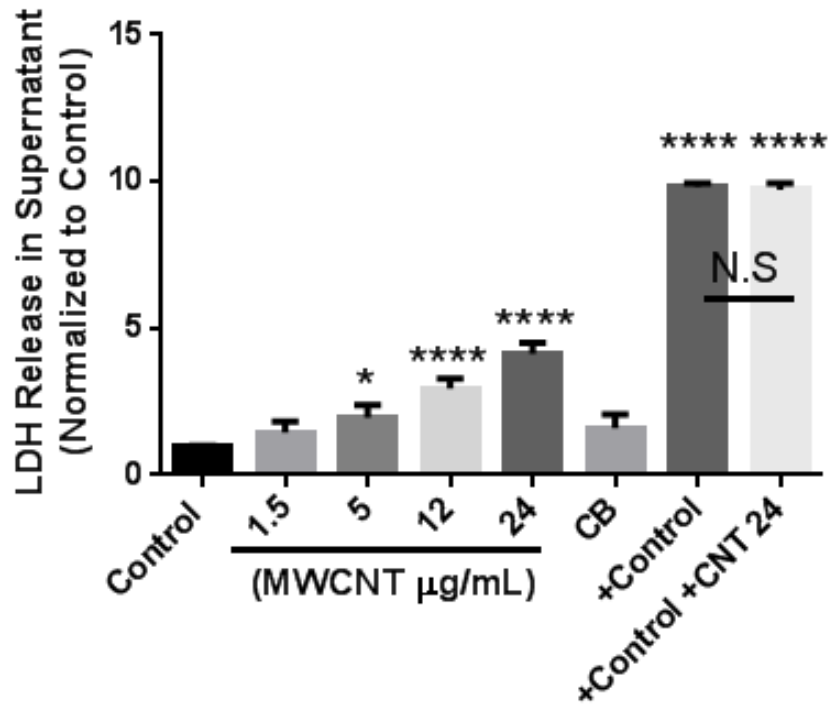

**B**

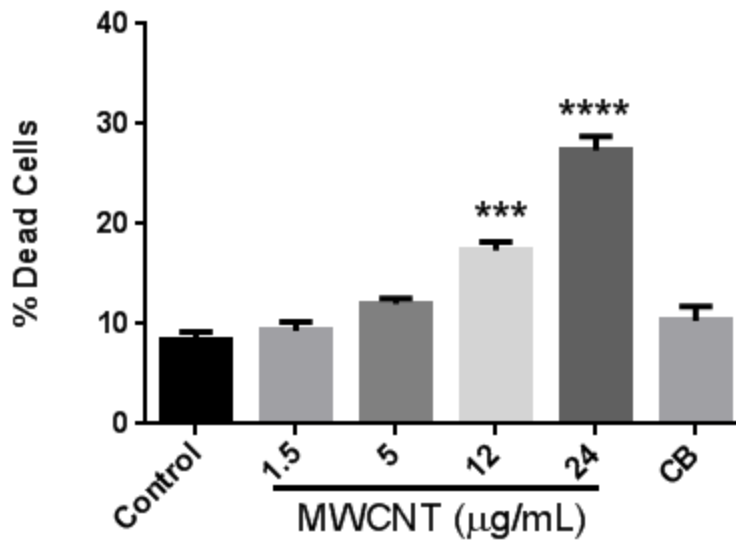

Supplement: Additional file 4 — LDH Release and Trypan Blue Exclusion counts for toxicity estimation. A) a dose response of LDH release in the cell culture supernatants after MWCNT exposure for 24 hours. LDH positive control (1:5000) dilution was incubated with nanotubes (24 μg/ml) for 24 hours and measured to check for interference with LDH in addition to using particle only controls for absorbance artifact. B) trypan blue exclusion counts. Data were analyzed by analysis of variance (ANOVA) followed by Tukey’s post hoc test. Graphs show average ± SEM of three independent experiments with triplicate of each condition, *p < 0.05, ***p < 0.001, ****p < 0.0001 (between media-treated control and treatments). [file 1743-8977-11-28-S4.pdf]

## Additional File 5

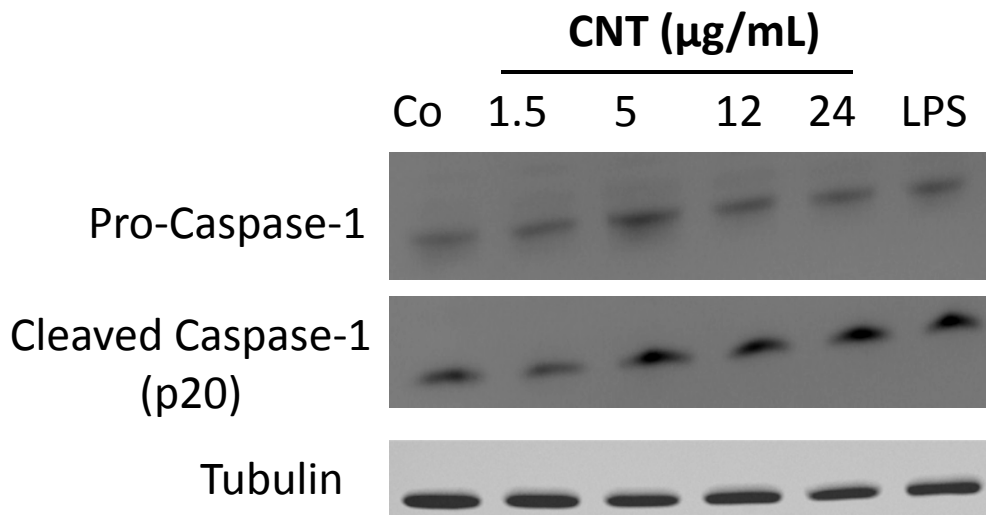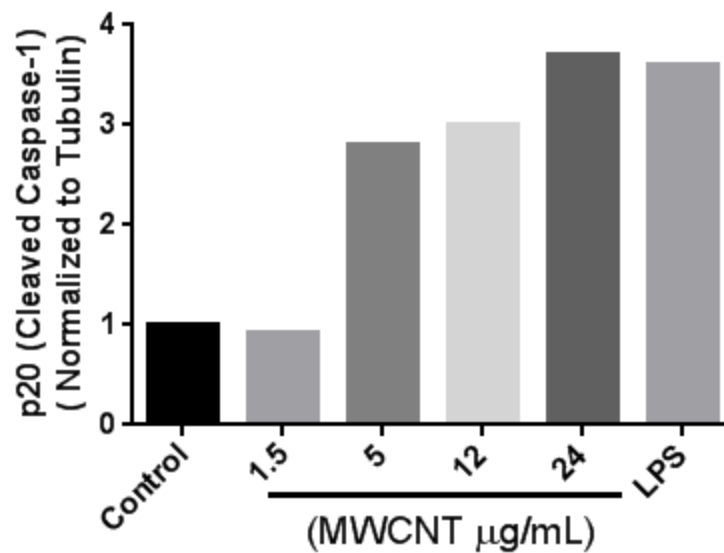

Supplement: Additional file 5 — Dose response analysis of caspase-1 (total and cleaved/active form) in HBE cells after MWCNT exposure for 24 hours. Tubulin was used as loading control. [file 1743-8977-11-28-S5.pdf]

## Additional File 6

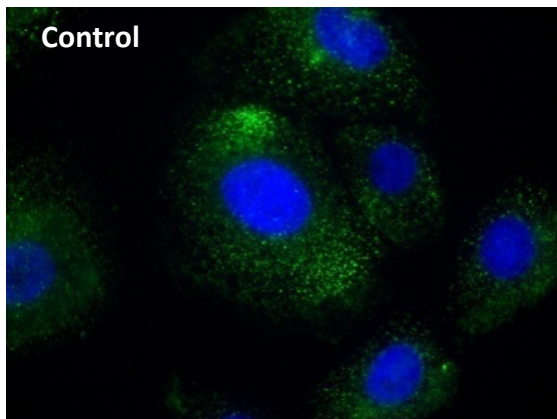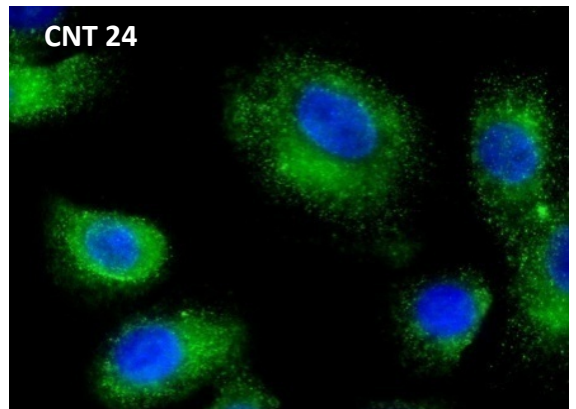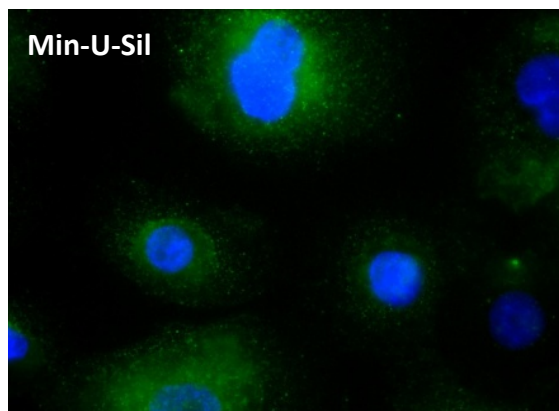

**Green : Cathepsin B**

**Blue : Hoechst (Nuclei)**

Supplement: Additional file 6 — Immunocytochemistry for Cathepsin B in HBE cells after exposure to MWCNT (24 μg/mL) for 24 hours. Nuclei were counter stained with Hoechst. Images are representative of 3 independent experiments done in duplicate. [file 1743-8977-11-28-S6.pdf]

# Additional File 7

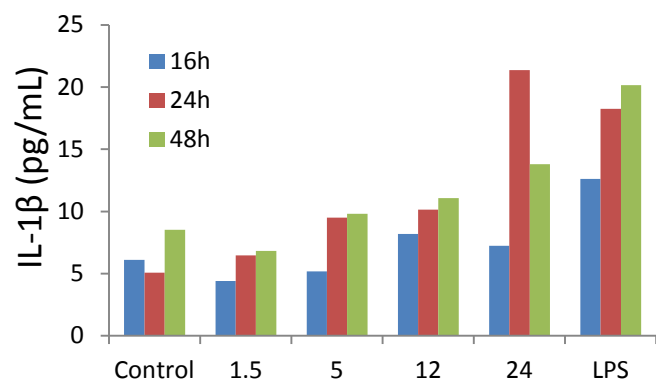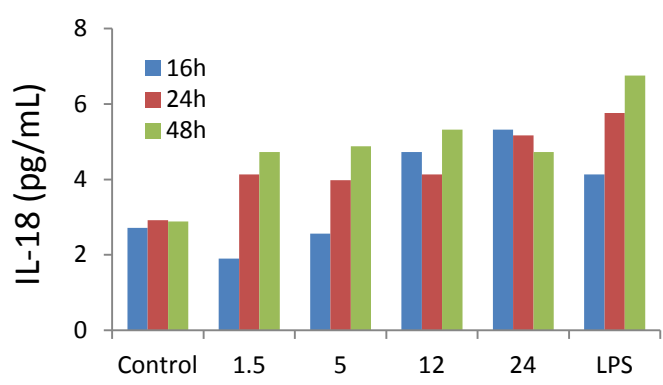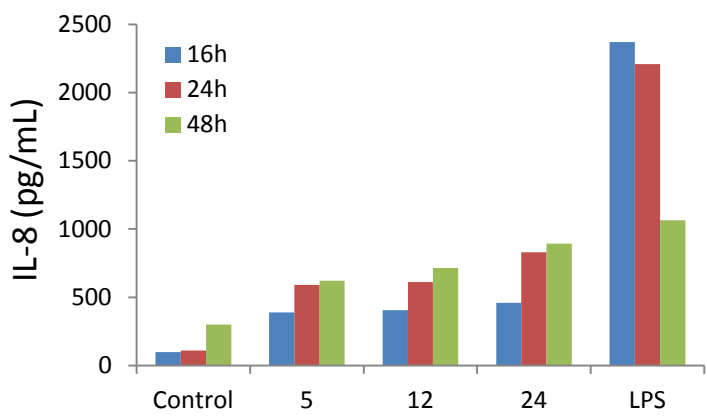

Supplement: Additional file 7 — Time course for inflammatory cytokine production. [file 1743-8977-11-28-S7.pdf]

# Additional File 8

A

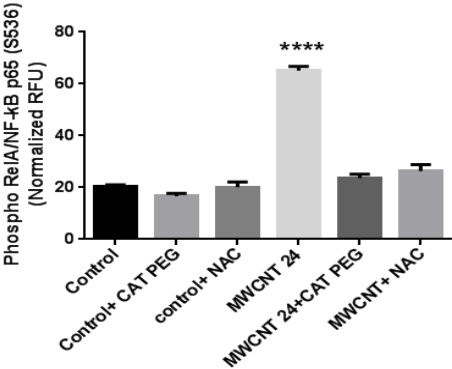

B

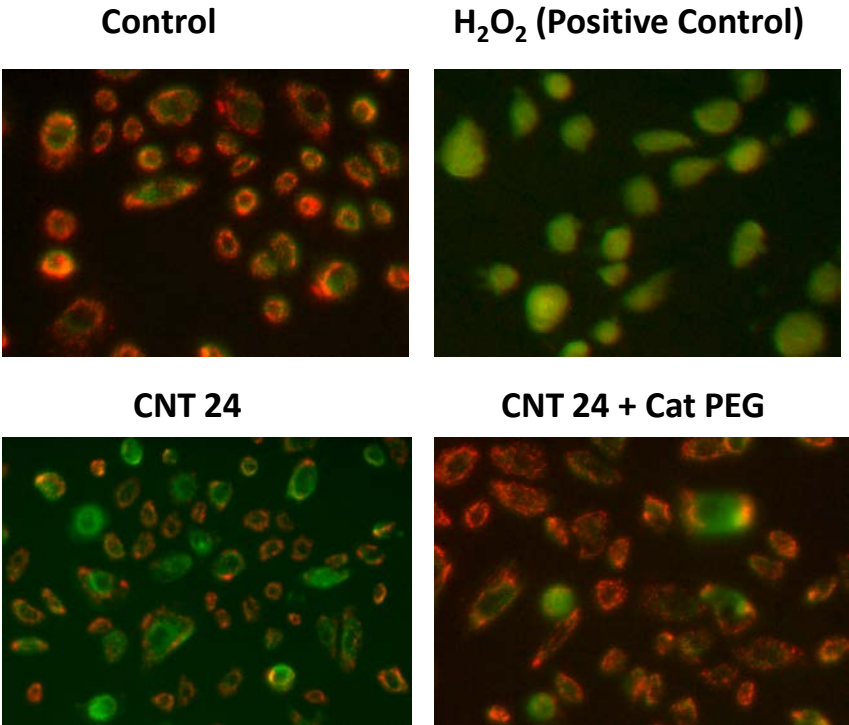

C

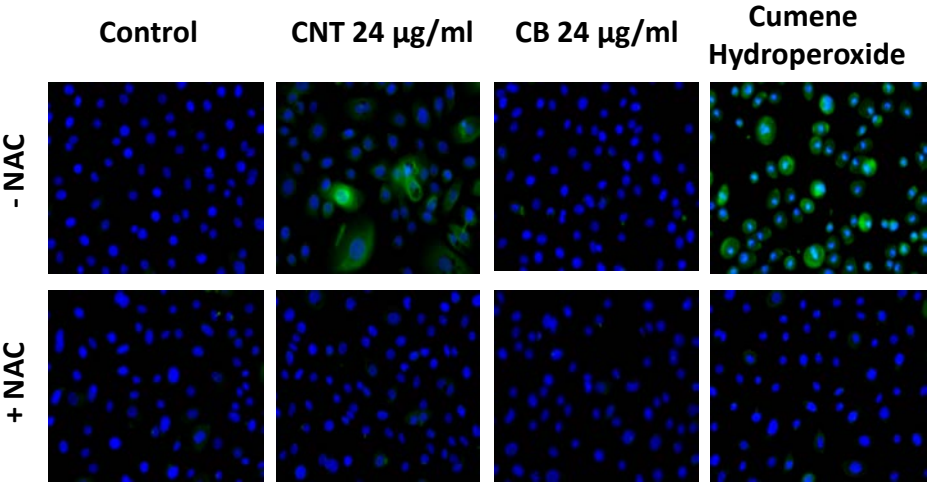

Supplement: Additional file 8 — A) Modulation of NF-κB (p65) phosphorylation, B) mitochondrial membrane potential changes and C) lipid peroxidation assessment using antioxidants (NAC and Cat Peg). Data were analyzed by analysis of variance (ANOVA) followed by Tukey’s post hoc test. Graphs show average ± SEM of three independent experiments with triplicate of each condition, ****p < 0.0001(between media-treated control and treatments). Images are representative of 3 independent experiments done in duplicate. [file 1743-8977-11-28-S8.pdf]

# Additional File 9

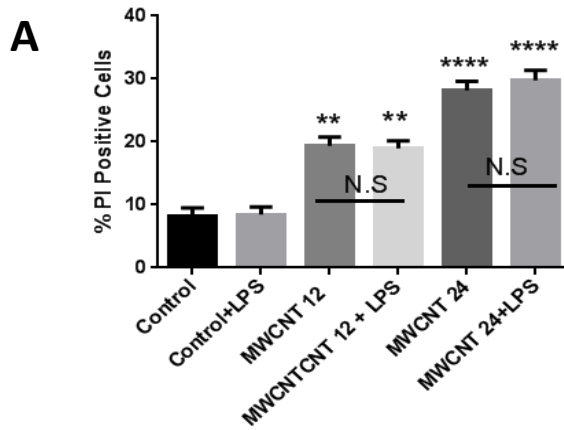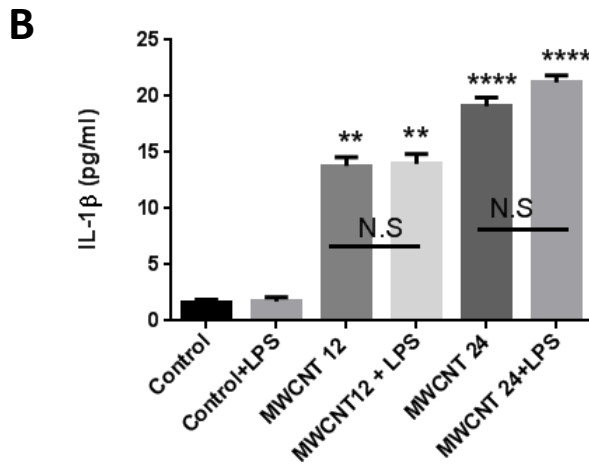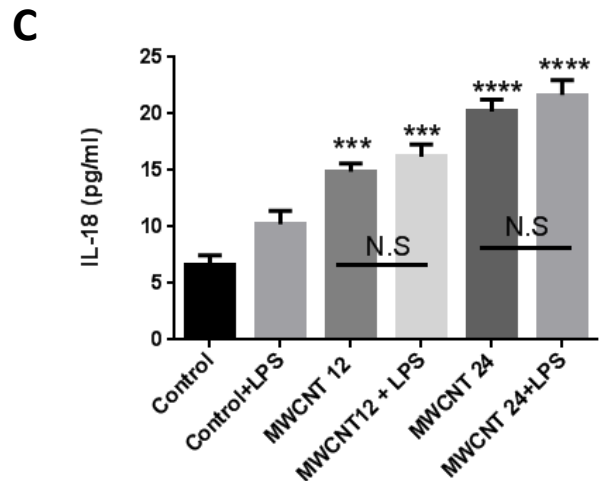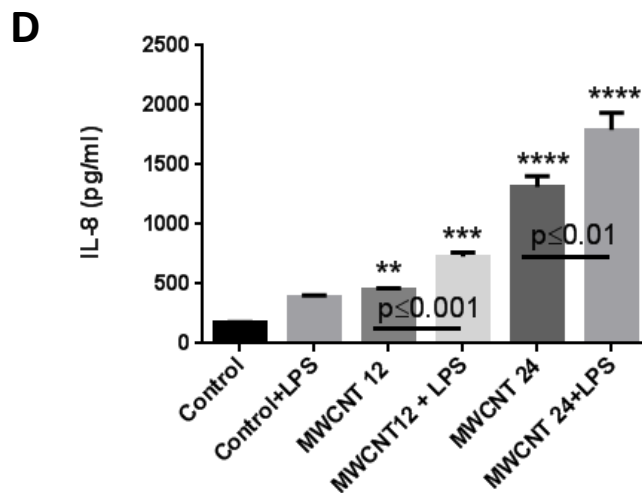

Supplement: Additional file 9 — Modulation of cytotoxicity and inflammation after LPS pre-stimulation of HBE cells. Cells were pre-stimulated with 1 μg/mL LPS for 2 hours and then treated with MWCNT (12 or 24 μg/mL) for 24 hours. A) cytotoxicity analysis by PI labelling followed by flow cytometery. B-D) inflammatory cytokine (IL-1β, IL-18, IL-8) production measured by ELISA. Data were analyzed by analysis of variance (ANOVA) followed by Tukey’s post hoc test. Graphs show average ± SEM of three independent experiments with triplicate of each condition, **p < 0.01, ****p < 0.0001 (between media-treated control and treatments). [file 1743-8977-11-28-S9.pdf]

## Additional File 10

**A**

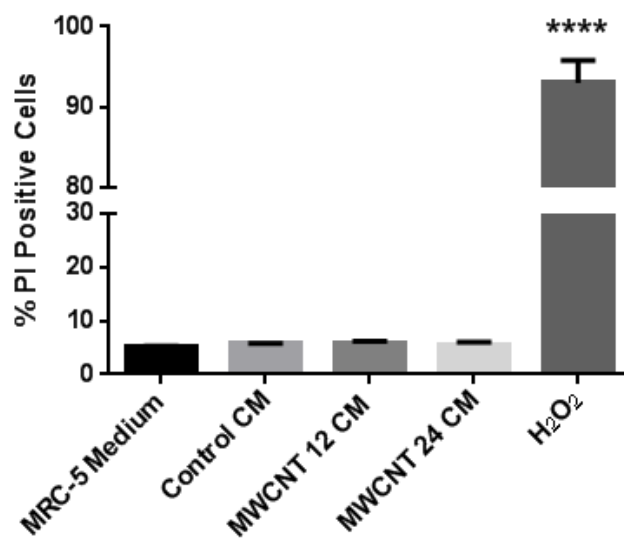

**B**

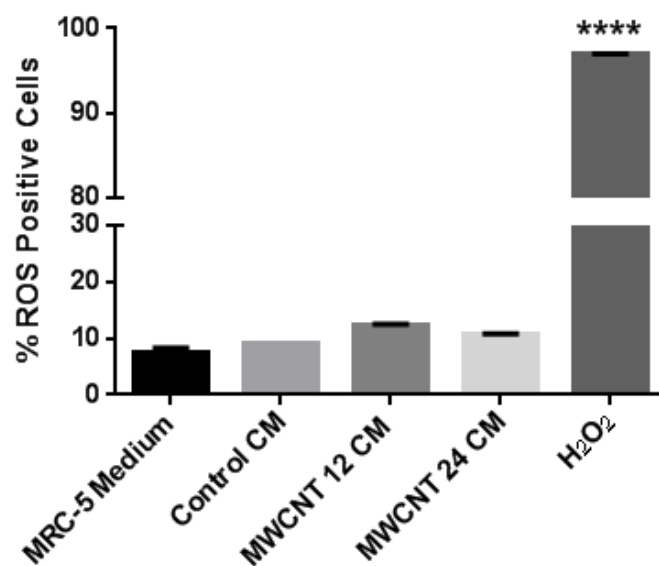

Supplement: Additional file 10 — Effects of MWCNT treated HBE cell conditioned medium on A) cytotoxicity (PI analysis) and 2) ROS production (DHE analysis) from fibroblasts using flow cytometry. MRC-5 medium (DMEM/F12) alone was also used in parallel to know the non-specific effects of conditioned medium. Data were analyzed by analysis of variance (ANOVA) followed by Tukey’s post hoc test. Graphs show average ± SEM of three independent experiments with triplicate of each condition, ****p < 0.0001 (between media-treated control and treatments). [file 1743-8977-11-28-S10.pdf]

## Additional File 11

**A**

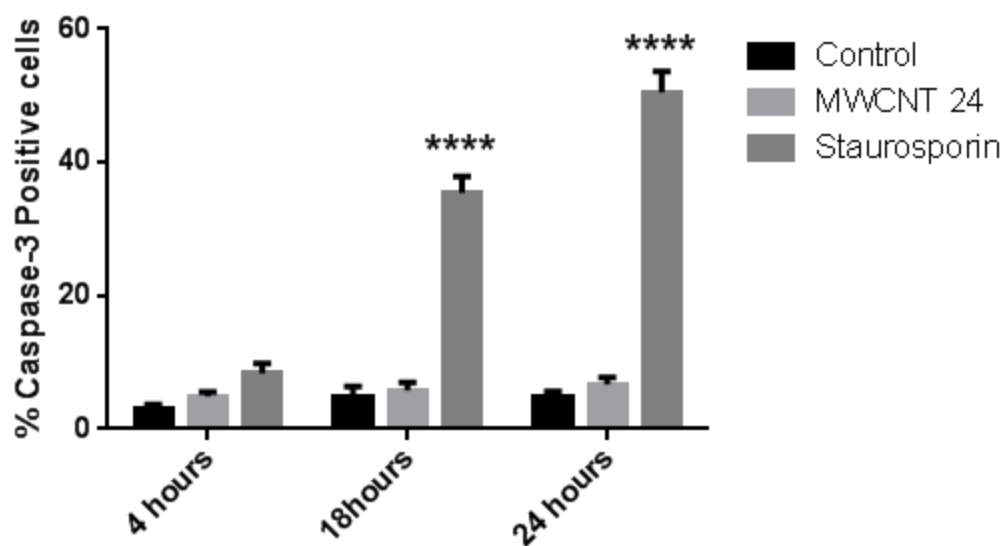

**B**

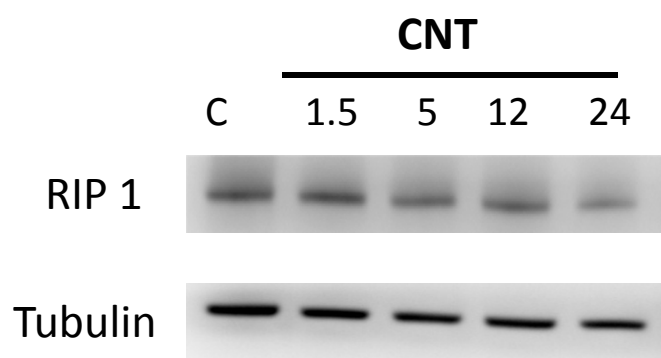

Supplement: Additional file 11 — Analysis of other modalities (apoptosis and necroptosis) of cell death. A) A time course analysis for caspase 3/7 activation after MWCNT exposure using flow cytometery. Staurosporine (10 μM) was used as a positive control. B) Western blot analysis for necroptosis protein RIP1. Data were analyzed by analysis of variance (ANOVA) followed by Tukey’s post hoc test. Graphs show average ± SEM of three independent experiments with triplicate of each condition, ****p < 0.0001 (between media-treated control and treatments). [file 1743-8977-11-28-S11.pdf]

## Additional File 12

### A) PI analysis

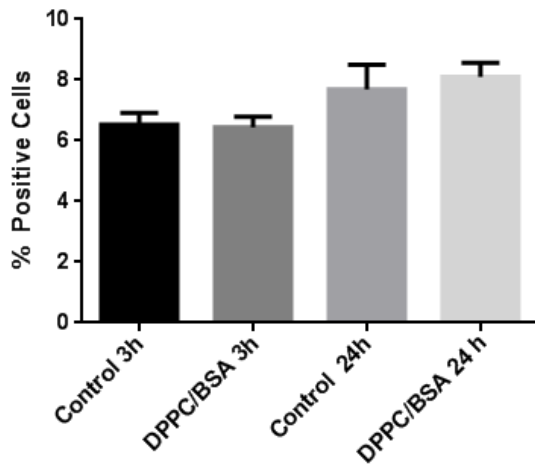

### B) Metabolic activity (WST-1)

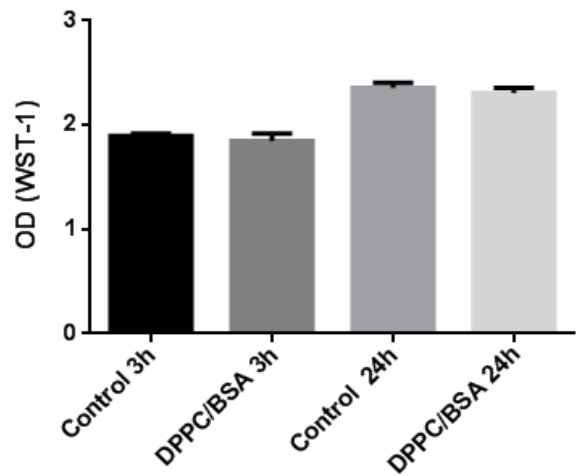

### C) Inflammation

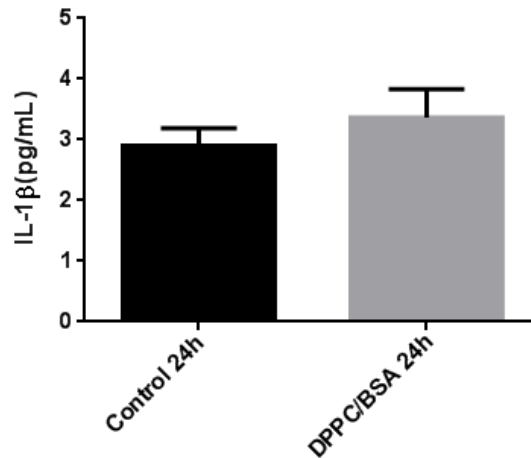

Supplement: Additional file 12 — Effect of media dispersants (BSA/DPPC) on the A) toxicity B) metabolic activity C) IL-1β production by HBE cells. [file 1743-8977-11-28-S12.pdf]

## Additional File 13

**A**

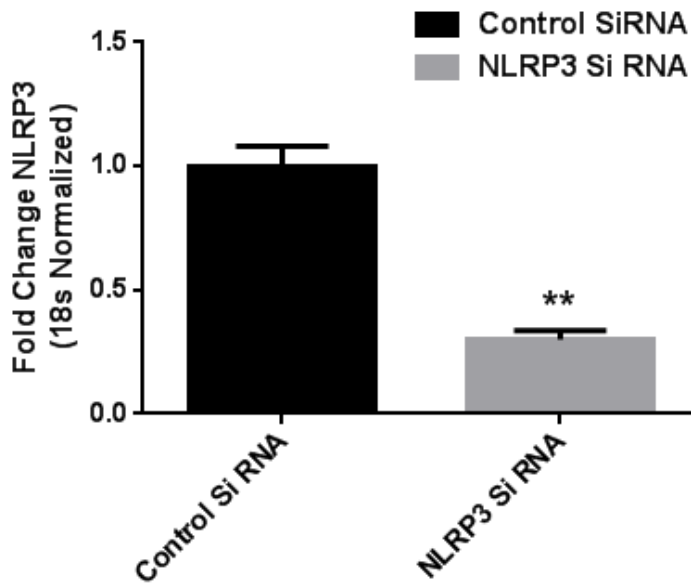

**B**

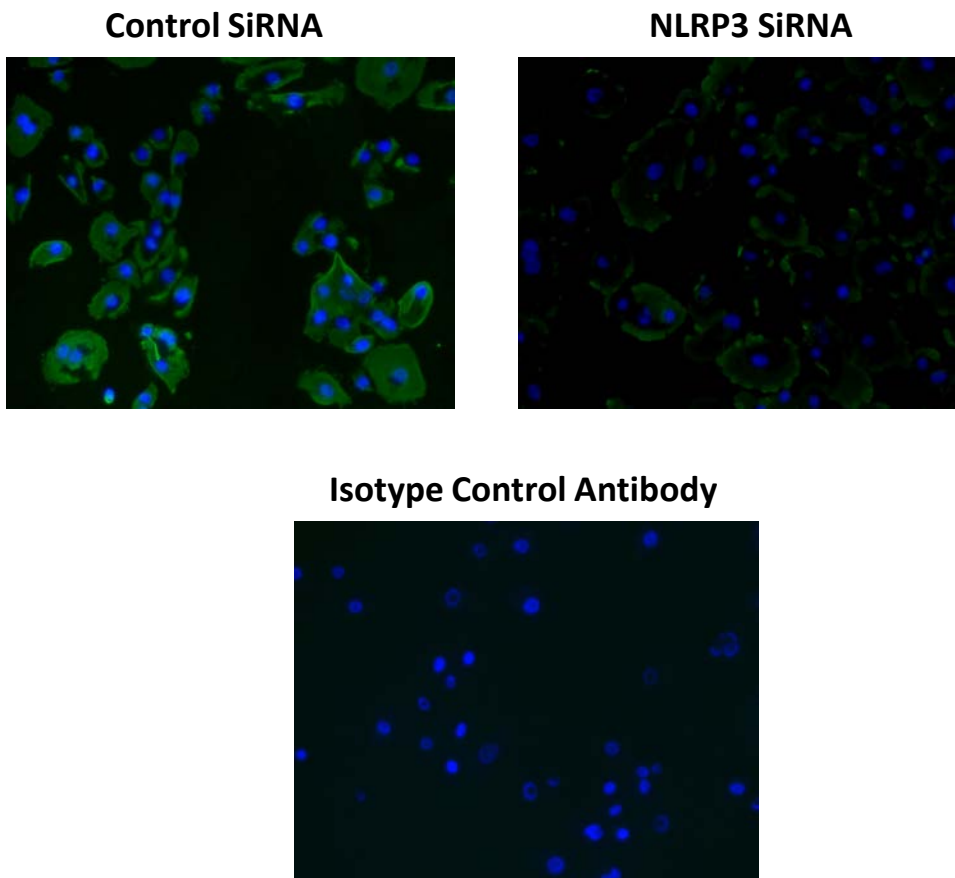

Supplement: Additional file 13 — Efficiency of NLRP3 SiRNA knockout A) real time RT-qPCR analysis B) immunostaining of HBE cells with our without NLRP3 SiRNA and with isotype control antibody. Graph shows average ± SEM of three independent experiments with triplicate of each condition, **p < 0.01 (between control SiRNA and NLRP3 SiRNA, Students t-test). [file 1743-8977-11-28-S13.pdf]
